# Proteome Turnover in the Spotlight: Approaches, Applications, and Perspectives

**DOI:** 10.1074/mcp.R120.002190

**Published:** 2020-12-07

**Authors:** Alison Barbara Ross, Julian David Langer, Marko Jovanovic

**Affiliations:** 1 Department of Biological Sciences, Columbia University, New York, New York, USA; 2 Proteomics, Max Planck Institute of Biophysics, Frankfurt am Main, Germany; 3 Proteomics, Max Planck Institute for Brain Research, Frankfurt am Main, Germany

**Keywords:** Proteome turnover, Mass spectrometry, Proteomics, Protein degradation, Protein synthesis, Dynamic SILAC, pSILAC, AHA, azido-homo-alanine (methionine analog), BONCAT, bio-orthogonal non-canonical amino acid tagging, LC-MS/MS, liquid chromatography coupled to tandem mass spectrometry, PECA, protein expression control analysis, pSILAC, pulsed stable isotope labeling of amino acids in cell culture, SILAC, stable isotope labeling of amino acids in cell culture, SRM, selected reaction monitoring, TMT, tandem mass tags, UPS, ubiquitin–proteasome system

## Abstract

In all cells, proteins are continuously synthesized and degraded to maintain protein homeostasis and modify gene expression levels in response to stimuli. Collectively, the processes of protein synthesis and degradation are referred to as protein turnover. At a steady state, protein turnover is constant to maintain protein homeostasis, but in dynamic responses, proteins change their rates of synthesis and degradation to adjust their proteomes to internal or external stimuli. Thus, probing the kinetics and dynamics of protein turnover lends insight into how cells regulate essential processes such as growth, differentiation, and stress response. Here, we outline historical and current approaches to measuring the kinetics of protein turnover on a proteome-wide scale in both steady-state and dynamic systems, with an emphasis on metabolic tracing using stable isotope–labeled amino acids. We highlight important considerations for designing proteome turnover experiments, key biological findings regarding the conserved principles of proteome turnover regulation, and future perspectives for both technological and biological investigation.

## Highlights

• Cellular proteins are in constant flux of synthesis and degradation.
• Mass spectrometry enables comprehensive, quantitative analyses of proteome turnover.
• Several labeling strategies provide direct proteome turnover measurements.
• Proteome turnover studies have answered a variety of important biological questions.

## Graphical Abstract

**Figure:**
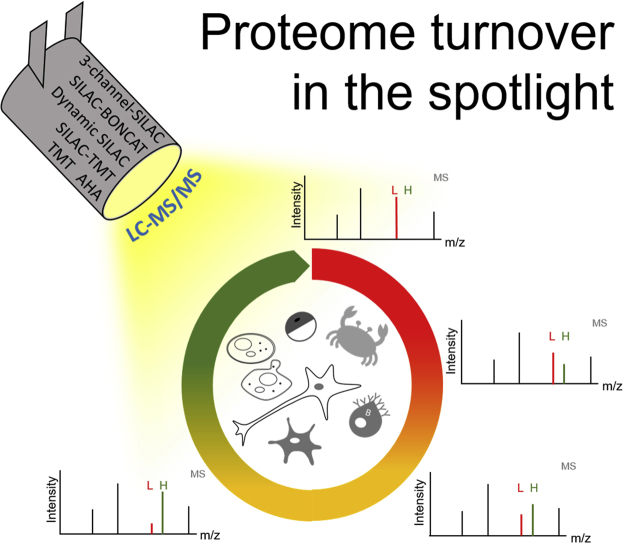

## In Brief

In this review, we outline historical and current approaches to measure the kinetics of protein turnover on a proteome-wide scale in both steady-state and dynamic systems, with an emphasis on metabolic tracing using stable isotope–labeled amino acids. In addition, we highlight important considerations for designing proteome turnover experiments, key biological findings regarding the conserved principles of proteome turnover regulation, and future perspectives for both technological and biological investigations.

In all cells, proteins are continuously produced and degraded, a process referred to as protein turnover. Protein turnover is regulated by several tightly controlled processes that help facilitate protein homoeostasis, also known as proteostasis (1, 2, 3, 4, 5, 6, 7). Proteostatic mechanisms are some of the cell’s most essential processes, as they ensure that functional proteins are maintained at their correct concentrations and in the proper locations needed for cellular activities to proceed (8, 9, 10). These processes also ensure that misfolded, aged, or damaged proteins are removed from the cellular protein pool as needed (11). Accordingly, disruption of proteostasis contributes to the pathophysiology of a variety of disease states, most notably neurodegenerative disorders and cancer (11). Probing the kinetics of proteome-wide protein turnover lends insight into how cells perform crucial functions such as differentiation and stress response in both normal and disease contexts, and can illuminate the guiding principles that underlie the regulation of protein turnover across protein families, cell types, and species.

Protein turnover is monitored and regulated by several cellular surveillance systems. Although protein production includes all of the processes that precede mRNA translation, including RNA transcription, maturation, and processing, in this review, we will focus on the time frame between protein synthesis and degradation. mRNA translation is controlled by regulatory motifs in mRNA nucleotide sequences; these sequences are bound by RNA-binding proteins and small RNA guides (such as microRNAs) to modulate their expression (12, 13, 14, 15, 16). Molecular chaperones, insertases, and translocases control maturation of nascent polypeptide chains, and post-translational modifications are added to proteins in the secretory pathway or through signaling cascades (17, 18, 19). Protein degradation occurs via two proteolytic machineries: the lysosome and the proteasome. The ubiquitin–proteasome system (UPS) is the main pathway for selective protein degradation, which uses a diverse collection of E1, E2, and E3 ubiquitin ligases to add ubiquitin to both cytosolic and nuclear proteins, targeting them for degradation by the proteasome (20, 21). In lysosomal proteolysis, proteins are engulfed by membrane-enclosed vesicles, such as autophagosomes or endocytotic vesicles, which then fuse with the membrane-enclosed lysosome. The lysosome then degrades proteins through its endogenous digestive enzymes (22).

When cells are perturbed, they change expression levels of specific proteins to respond to their new requirements and adjust their cellular functions accordingly. Historically, proteome studies quantified protein abundances to track differentially expressed proteins in various cell states. Technical and methodological developments in the past 15 years, however, now enable researchers to specifically monitor protein synthesis and degradation on a proteome-wide level and in dynamic systems. By providing insight into the processes by which cells maintain and dynamically adjust their proteomes to suit their needs, these emerging technologies can provide another dimension of information to quantitative proteomics studies. This information has already yielded important new insights into the molecular mechanisms involved in cellular protein homeostasis during physiological processes such as cellular differentiation, various neuronal functions, and the immune response, which we will highlight throughout this review.

Today’s high-throughput methods for analyzing proteome turnover are extensions of decades of previous biochemical and biophysical investigations. Both modern and historical metabolic turnover and protein half-life measurements are based on the so-called “pulse” approach, which requires the introduction of radioactive, biochemical, or stable isotope–labeled tracers into target proteins. These tracers are introduced into the target cell’s metabolism either through the solvent (*e.g.*, H_2_O and hydrogen isotopes), carbon, or nitrogen metabolism (*e.g.*, ^13^C-labeled carbohydrates or ^15^N-labeled ammonia salts), or complete amino acids (*e.g.*, ^13^C_6_-lysine or ^13^C_6_^15^N_4_-arginine). Tracers can then be monitored using a corresponding detection system (23). In the “pulse-chase” paradigm, the “pulse” is followed by a “chase” period, in which the labeled tracer is replaced—“chased” away—by an excess of the same unlabeled compound after a certain period of time. Depending on the experimental setup, following the labeled tracer over time measures its incorporation (protein production) and/or its loss (protein degradation). We will primarily focus on the use of amino acid–based tracers coupled to mass spectrometry–based proteomics to determine the turnover rates of hundreds to thousands of proteins, but we will briefly outline alternative and complementary approaches, as well.

## Definition of Terms and Turnover Rate Modeling

### General Assumptions About Synthesis and Degradation Rates

**Fig. 1:**
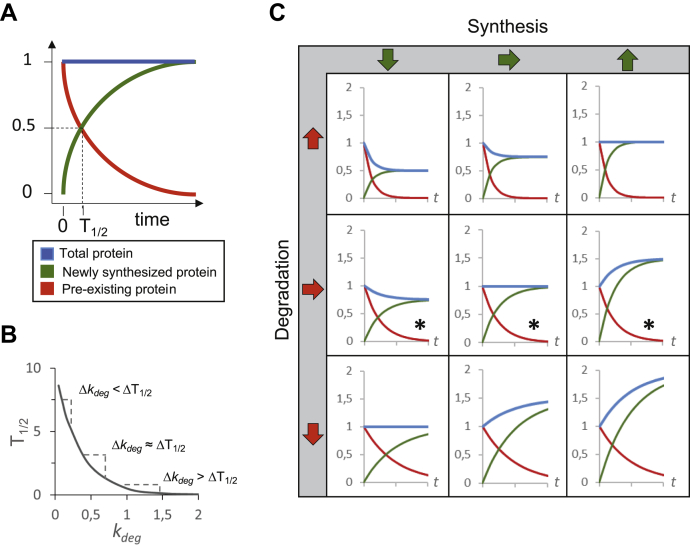
**Schematic plots illustrating the correlations and interdependencies found between protein turnover parameters**. *A*, increase of proteome-wide newly synthesized protein (*green*) and decay of pre-existing protein (*red*) plotted over time at steady state, with constant total protein levels indicated in *blue*. Protein half-life (T_1/2_) is indicated by *dashed lines*. *B*, the plot of the general relationship between half-life (T_1/2_) and degradation rate constant (*k*_deg_): T_1/2_ = ln(2)/*k*_deg_. Note that small changes at low *k*_deg_ values lead to more pronounced changes in T_1/2_ than at high *k*_deg_ values. *C*, the schemes of interplay between synthesis, degradation, and total protein amounts in different exemplary conditions: if synthesis is reduced (*first column*), stays constant (*middle column*), or is increased (*right column*), and if degradation is increased (*top row*), stays constant (*middle row*), or is decreased (*bottom row*). Effects on total protein amounts schematically represented by *blue lines*. The *asterisk* indicates constant *k*_deg_ and therefore equal protein turnover, although the absolute protein amount could change because of protein synthesis rate changes.

We will first define a few key terms used to describe proteome turnover. In general, cellular protein amount is determined by the rate at which that protein is synthesized and degraded (Fig. 1*A*). Inherently, synthesis rates and degradation rates differ in their mathematical properties. This is due to the fact that synthesis is a zero-order process whose rate of change is measured in units of protein amount over time; accordingly, the synthesis rate constant *k*_syn_ can be expressed in units of moles/time. Degradation, on the other hand, is a first-order process whose rate corresponds to the fractional removal of proteins from an existing pool in the cell. The degradation rate constant *k*_deg_ is therefore quantified with the dimension of time only (1/time). As the amount of protein decreases at a rate (*k*_deg_) proportional to its current value, the amount of protein lost follows an exponential decay function. As such, the amount of protein produced over a certain time window depends only on the integration of its synthesis rate constant, *k*_syn_:

(1) $$\text{dP}/\text{dt}\left(\text{syn}\right)=k_{\text{syn}}$$

while the amount of protein lost over that time frame depends on the existing protein pool multiplied by its degradation rate constant, *k*_deg_ (24):

(2) $$\text{dP}/\text{dt}\left(\text{deg}\right)=\left[\text{P}\right]\text{∗}k_{\text{deg}}$$

or if expressed as an exponential decay function:

(3) $$\text{P}\left(\text{t}\right)={\text{P}}_{0}*e^{-\text{k}\deg*\text{t}}$$

together Equations 1 and 2 define the total change in protein amount over time:

(4) $$\text{dP}/\text{dt}\left(\text{total}\right)=k_{\text{syn}}-\left[\text{P}\right]*k_{\deg}$$

### Turnover Rates in Steady-State *Versus* Dynamic Systems

In this review, we distinguish between cells at steady state and those undergoing a dynamic change. At steady state, we can make two basic assumptions (1): for all proteins, the net change in protein levels is zero, which means that (2) the number of protein molecules produced is equal to the number of proteins lost.

At steady state:

(5) $$\text{dP}/\text{dt}=k_{\text{syn}}-\left[\text{P}\right]*k_{\deg}=0$$

(6) $${\text{k}}_{\text{syn}}=\left[\text{P}\right]*k_{\deg}\to \left[\text{P}\right]=k_{\text{syn}}/k_{\deg}$$

The turnover rate of a protein is often defined as the time needed to both degrade and resynthesize half the proteins present in a specific cellular state. At steady state, however, owing to the equivalency described in Equation 6, the turnover rate is simply equal to the time it takes to remove half of the existing protein pool—and as such, relative turnover rates can simply be expressed through the degradation rate constant *k*_deg_. Oftentimes, relative turnover rates are also defined in terms of half-life (T_1/2_), which is simply a reciprocal derivative of *k*_deg_ (24) (Fig. 1*B*):

(7) $${\text{T}}_{1/2}=\ln\left(2\right)/k_{\deg}\text{.}$$

During dynamic processes, protein levels often change over time. Protein-level changes may be due to changes in protein production rates, protein degradation rates, or both (Fig. 1*C*). When a new steady state is reached after the perturbation, a protein may be expressed at a very different abundance than before, but its turnover rate will only differ if its degradation rate constant, *k*_deg_, and therefore its half-life, has changed. In other words, based on the definitions above, protein synthesis changes alone will not affect a protein’s turnover rate, but only its abundance (Fig. 1*C*).

Modeling true changes in turnover rates during dynamic processes requires considerably more mathematical manipulation than modeling turnover rates at a steady state (25)—in fact, achieving an accurate model of dynamic turnover rate changes remains an open challenge in the field. So far, dynamic changes in turnover rate constants have only been approximated using linear rate change assumptions, which do not likely fully represent the true physiological behavior of dynamically adjusting proteomes (25). However, the above steady-state assumptions can and have been used effectively to compare relative end-point synthesis and degradation rates between conditions as we will describe below.

### “Old” Proteins and Non-exponential Decay

Different methods described in the text below each confer particular advantages and disadvantages for tracking protein degradation and synthesis rates at steady state (supplemental Table S1). Modeling turnover rates according to the definitions at steady state described above is relatively straightforward, with one notable exception. We assume that the first-order process of protein loss is stochastic and all proteins from the same species have the same probability of getting degraded. Under this assumption, a newly synthesized protein has the same probability of being degraded as a pre-existing proteins synthesized much earlier (24), and as such, protein loss follows an exponential decay function as described in Equation 3. This also explains why the synthesis signal from metabolic labels appears logarithmic rather than linear, despite protein synthesis following zero-order kinetics as described in Equation 1 (Fig. 1). However, recent studies have demonstrated that the assumption of exponential decay does not hold true for all cellular protein populations. For certain subsets of proteins, the probability that any given protein molecule is degraded can change as a function of its molecular age, with newly synthesized proteins being typically less stable than “older” proteins. The loss of these proteins follows a pattern of non-exponential behaviors (see the text below for more details) (26, 27, 28, 29).

### Cell Division and Protein Turnover

As outlined above, the critical protein turnover parameter, *k*_deg_, corresponds to the time it takes for a cell’s pre-existing protein pool to be reduced by half. This is certainly true for nondividing cells, but for dividing cells, the pre-existing protein pool will be reduced to half with every cell division even without active protein degradation. In dividing cells, therefore, the reduction of a pre-existing protein pool occurs because of a combination of dilution due to cell division and true protein degradation. In such a system, the cell division rate must be taken into account and should be included as its own term, *k*_dil_. The total rate of protein loss, *k*_loss_, measured in such a system is defined by the following equation:

(8) $$k_{\text{loss}}=k_{\text{dil}}+k_{\deg}$$

Consequently, *k*_deg_ can be determined by measuring *k*_loss_ and the division rate, *k*_dil_, of the studied system. Taking the division rate into account is extremely important, not just for comparing systems for which cell division rates vary greatly, but for accurately detecting the *k*_deg_ of proteins that turn over slowly, which can be confounded by *k*_dil_ (24). More details about the non-trivial relationship between protein synthesis, degradation rate, and cell division rate are discussed in depth in a recent publication by the Busse group, who emphasize that taking the cell division rate into account significantly improves the “hit rate” of differential gene expression profiling (30).

## Radioactivity, Drugs, and Fluorescent Lights—Even Before the 70s

In the first protein turnover studies more than 80 years ago, ^15^N-isotope–labeled amino acids were fed to mice to analyze protein synthesis and degradation, with detection based on mass spectrometry (2, 3, 4, 5, 6). These groundbreaking studies showed that cellular proteins are not static but rather are in constant flux of production and loss. In the following decades, the most commonly used reporters were amino acids with radioactive isotopes of carbon, hydrogen, or sulfur, with subsequent detection in proteins using scintillation counting (31). Radioactive decay–based detection of synthesis and degradation of specific proteins enabled direct analyses of their half-lives, particularly in combination with antibody-based purification of the target proteins (32). Later on, the combination of autoradiography with 1D and 2D gels allowed for more comprehensive differential turnover studies, as dozens of different proteins can be separated on such gels (33, 34). However, these analyses were limited by the relatively high radioactivity doses required (and their associated effects on cell and animal health), the significant protein loss during sample preparation, and the challenging identification of candidate proteins displaying differential synthesis or decay kinetics (35).

Subsequently, the application of biochemical tools, such as small-molecule inhibitors of synthesis (*e.g.*, cycloheximide, puromycin) or degradation (*e.g.*, MG-132, bortezomib, lactacystin), alongside the invention of genetically engineered proteins, enabled new insights into proteome stability and turnover. Fusion proteins tagged with constructs such as GFP or the tandem affinity purification tag allowed for comprehensive, highly multiplexed studies with specific and sensitive detection of protein synthesis and degradation; with these tags, no introduction of tracer amino acids was required (36, 37, 38). For example, a systematic study in yeast, performed in the year 2006, reported degradation rates for more than 3750 tandem affinity purification–tagged proteins after inhibition of protein synthesis by cycloheximide, with detection of protein loss over time determined by immunoblotting (36). Two years later, the stability of more than 8000 proteins was profiled in HEK293T cells using a combination of GFP tagging, flow cytometry, and microarrays (37). These studies all required the construction of thousands of reporter-tagged strain or cell line collections—a mammoth task to accomplish in both resources and manpower.

Technical advances in mass spectrometry–based proteomics then allowed for more generalized protein tracing, without the need for tagged constructs. In combination with translation inhibition, shotgun proteomics facilitated the tracking of protein degradation rates. This approach has been applied alongside subcellular fractionation and proteasome inhibition to quantify the differences in subcellular proteome turnover and match degradation pathways to each cellular compartment (39).

Although all of these tools provided valuable new insights, they incur considerable limitations (supplemental Table S1). First, the inhibition of protein synthesis or degradation (*e.g.*, by drugs) may lead to compensatory and off-target effects that can make determining physiological turnover rates challenging (40). Second, protein tags can potentially compromise physiological protein function and half-life. This is particularly critical for small proteins and membrane proteins (41, 42, 43). Third, the construction of large libraries of tagged protein constructs is both time and resource intensive. Nevertheless, these approaches are still widely used, particularly in targeted studies exclusively examining either protein synthesis or degradation. They are also still commonly used for specific and sensitive detection of a particular protein of interest with techniques such as Western blotting or immunofluorescent imaging to determine subcellular localization.

## Dynamic SILAC Approaches

In the early 21st century, the use of nonradioactive isotopes in combination with mass spectrometry became popular in proteome turnover studies. The combination of high-resolution liquid chromatography, nanoelectrospray ionization, and ultrahigh-resolution tandem mass spectrometry with fast MS/MS cycles enabled the quantitative analysis of thousands of peptides and proteins in a few hours. The required tracer isotopes were initially introduced via carbon sources (44), in which heavy isotopes were incorporated into proteins by sugar/carbon metabolism. However, the incorporation of heavy isotopes via metabolic pathways normally leads to a variation in the degree to which heavy-labeled amino acids were incorporated into proteins, such that full labeling was often not achieved. This made the separation of overlapping isotopic envelopes, and therefore quantification of the differently labeled peptides, very challenging (1, 44, 45, 46).

This was largely overcome by the addition of amino acids with a defined and selectable number of stable isotopes into culture media or food sources, allowing for comprehensive and systematic proteome turnover studies in a variety of organisms (47). These “heavy” amino acids were initially used for quantitative studies as part of stable isotope labeling by amino acids in cell culture (stable isotope labeling of amino acids in cell culture [SILAC]), in which proteome abundance differences in unlabeled and fully labeled samples are compared (48, 49). Because specific amino acids—normally lysine and/or arginine—were labeled with a fixed mass in SILAC, the isotopic envelopes of “light” and “heavy” peaks are separated by predefined shifts (*e.g.*, ^13^C_6_-lysine or ^13^C_6_^15^N_4_-arginine), greatly facilitating data acquisition and interpretation. Coupling lysine/arginine labeling with trypsin as a protease for sample preparation guarantees that nearly each peptide has a labeled amino acid.

**Fig. 2:**
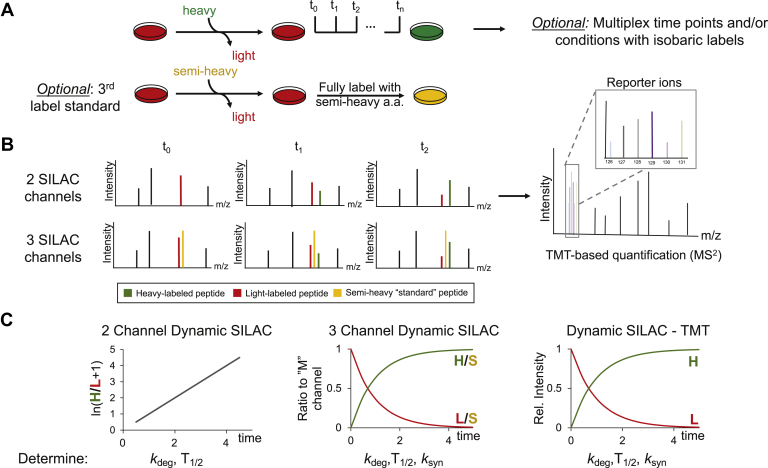
**The dynamic SILAC workflow**. *A*, sample preparation. In a standard two-label dynamic SILAC experiment, cultures are plated for example in an unlabeled media and then the media is swapped for the one containing stable isotope–labeled amino acids (*e.g.*, ^13^C_6_^15^N_4_-Arg–“heavy” arginine). Samples are then collected over a time course, with a separate culture harvested at each time point. After sample digestion and purification, isobaric labeling (*e.g.*, by TMT, as depicted) can be used to multiplex samples from multiple conditions and time points of interest. A fully labeled sample using a third stable isotope (*e.g.*, ^13^C_6_-Arg), typically a "semi-heavy /medium-heavy" isotope, can also be generated as normalization standard for data analysis. It should be noted that label switches can also be performed in a different way than depicted here (*e.g.*, cells good be grown in "heavy" amino acids and then pulsed with "light" amino acids). *B*, data acquisition. LC-MS/MS enables direct monitoring of "light" (*red*) and "heavy" (*green*) peptide signals, which correspond to pre-existing and newly synthesized proteins, respectively. For dynamic SILAC-TMT experiments, relative quantification of each sample is completed at the MS^2^ level (far right). In three-channel designs, the signal from the constant "semi-heavy"-labeled sample (*yellow*), spike-in provides an internal normalization standard between different mass spectrometry measurements, allowing for relative signal from "light" and "heavy" channels to be quantitated. *C*, data analysis. Here, we show data for an example protein measured from a two-channel dynamic SILAC experiment (left), a three-channel dynamic SILAC experiment (middle), and a combined two-channel dynamic SILAC-TMT experiment (right). With two-channel dynamic SILAC, half-lives and *k*_deg_ can be calculated using ln-transformed "heavy" over "light" (H/L) peak ratios over time, but owing to run-to-run variability during the mass spectrometry measurements, it is difficult to separate the contributions of synthesis and degradation. On the other hand, data from three-channel dynamic SILAC and dynamic SILAC-TMT can be used to determine *k*_syn_ separately from *k*_deg_. For three-channel dynamic SILAC, this can be achieved by plotting "heavy" isotope over "semi-heavy" isotope (H/S) signal to generate a synthesis curve, whereas plotting light-isotope over "semi-heavy" isotope signal (L/S) generates a curve for protein degradation. In dynamic SILAC-TMT, all the "heavy" (H) and "light" (L) signals are measured in the same run, which allows for separate synthesis and degradation curves. LC-MS/MS, liquid chromatography coupled to tandem mass spectrometry; SILAC, stable isotope labeling by amino acids in cell culture; TMT, tandem mass tag. SILAC, stable isotope labeling of amino acids in cell culture.

This quantitative approach was then repurposed to study proteome turnover by making use of “pulsed-only” experiments. In these so-called dynamic SILAC experiments, cells are switched from unlabeled medium to a medium containing isotopically labeled amino acids, still typically heavy lysine and/or arginine (50). Samples are then measured via liquid chromatography coupled to tandem mass spectrometry (LC-MS/MS) over a time course. The rate at which a heavy amino acid–labeled peptide signal appears corresponds mainly to that peptide’s rate of synthesis, whereas the rate at which a light amino acid–containing peptide decreases represents its rate of degradation. The ratio of heavy to light peptide signal thus directly reflects protein turnover (Fig. 2).

It should be noted here that dynamic SILAC and a similar term—pulsed SILAC (pSILAC)—are often used interchangeably in the literature. However, although both approaches are “pulsed-only” experiments with a similar setup, dynamic SILAC actually refers to experiments that determine proteome-wide protein turnover rates using only two SILAC channels, “light” and “heavy”. pSILAC, on the other hand, originally referred to a labeling approach in which two “light” cell populations are pulse-labeled with either “semi-heavy” or “heavy” amino acids to quantify relative differences in *de novo* protein synthesis. The term was first coined by Selbach *et al.* (51), who used pSILAC to assess the impact of microRNAs on protein synthesis, and subsequently described in more detail by Schwanhäusser *et al.* (52). Although the terms are now often used interchangeably, we will honor their original definitions and refer to dynamic SILAC in all studies that measured protein turnover (the majority of studies described here) and use pSILAC only in the manner that it was originally intended—to measure relative differences in *de novo* protein synthesis.

Although classical dynamic SILAC experiments have many advantages, they do have some limitations (supplemental Table S1). It should be noted that owing to the reuse of existing, light amino acids, the true synthesis rate of a protein may be higher than that measured by the increase in its heavy-labeled peak. This “recycling issue” can be addressed by monitoring peptides with missed cleavage sites and correcting for their uptake of light amino acids (53). In addition, because each sample over a time course must be harvested separately, it is difficult to make absolute comparisons of either the heavy or light isotope peak intensities over time because of experimental differences in sample preparation and data acquisition. In general, dynamic SILAC data yield protein turnover information (half-lives at steady state—see “Definition of Terms and Turnover Rate Modeling” section) but do not allow clear separation of the contribution of synthesis and degradation to the measured turnover rate without further internal standards or quantification strategies (Figure 2 and supplemental Table S1) (54, 55). Despite these limitations, dynamic SILAC approaches have provided valuable insights into cellular mechanisms of proteostasis in different cells, tissues, and diseases and can help elucidate the mechanisms by which cells, for example, differentiate, respond to perturbations, and perform their essential functions.

**Fig. 3:**
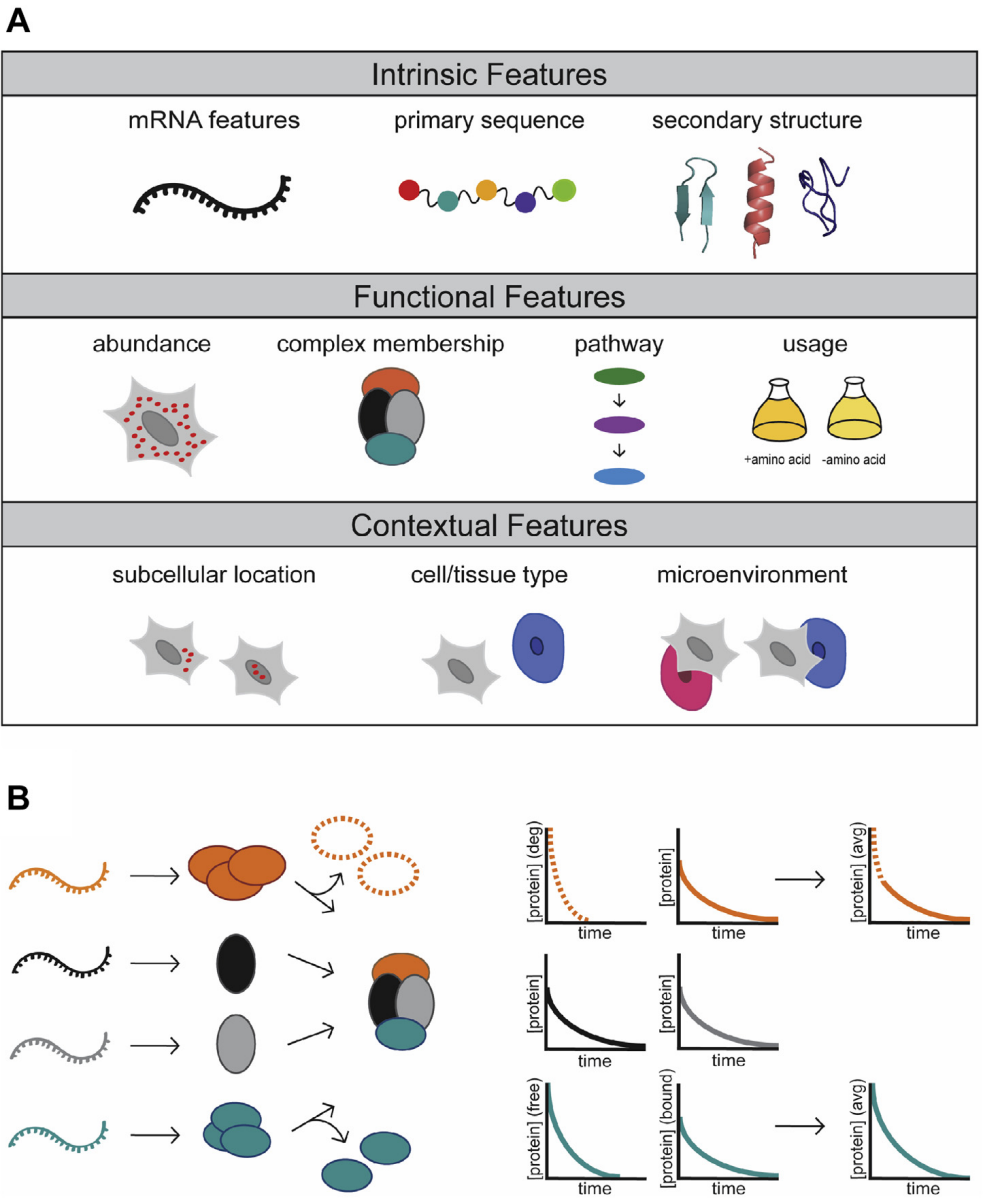
**Determinants of protein turnover rates**. *A*, several intrinsic, functional, and contextual parameters have been identified as possible determinants of protein turnover behaviors, with various degrees of consensus found within the published literature. Intrinsic features include mRNA features (mRNA half-lives and codon optimality), primary amino acid sequence features (presence of particular amino acids or motifs), and secondary structure features (alpha helices *versus* beta sheets *versus* disordered sequences). Functional features include relative abundance, complex membership and assembly, pathway relationships, and usage (such as shorter half-lives of proteins involved in biosynthesis of an amino acid after that amino acid is removed from the growth media). Contextual determinants include subcellular localization, cell and tissue type, and microenvironment (including presence of cell–cell interactions). *B*, proteome turnover studies have led to greater understanding of the dynamics of protein-complex synthesis, assembly, and degradation. Proteins involved in multimeric complexes tend to have turnover rates that are generally coherent. Although cells are capable of translating complex members at stoichiometric equivalencies, as has been shown in yeast and mammals, they can overexpress one or more members of the complex and then degrade a subset of them post-translationally to achieve stoichiometric equivalency (shown in *orange, dashed lines*). Some complex members may be synthesized in excess but not degraded and may perform additional functions either as free subunits or as members of other complexes (shown in *teal*). These proteins may actually have two different turnover rates depending on complex association or subcellular location, but only one aggregate turnover rate will be measured in standard dynamic SILAC-based approaches. Despite overall agreement in turnover rates, complex subunit half-lives show even greater coherency within subcomplex architecture (shown here in *gray* and *black*, with the two internal subunits showing the most similar degradation behavior). SILAC, stable isotope labeling by amino acids in cell culture.

The first dynamic SILAC study was performed by the Beynon lab in 2009, where they determined the turnover rate for nearly 600 proteins in human A549 adenocarcinoma cells and examined the intrinsic properties correlated with protein turnover (50). Soon thereafter, a seminal article showing the power of the dynamic SILAC approach was published in 2011 by the Selbach group (53). In this study, Schwanhausser *et al.* (53) measured both protein and mRNA turnover by metabolic labeling in unsynchronized, dividing NIH 3T3 mouse fibroblast cells. They found that mRNA levels and mRNA translation rates contributed the most to the final protein levels, whereas mRNA and protein stability only had a minor global effect. Moreover, they found that mRNA and protein turnover rates themselves showed no correlation to one another. This study illustrates one of the most straightforward applications of dynamic SILAC-based technology, which is to probe proteome-wide turnover rates and then match these rates with bioinformatic analysis to other parameters (*e.g.*, primary sequence, motifs, mRNA turnover, etc.) to identify the generalizable parameters that determine turnover rates under the measured conditions (Fig. 3*A*). More recently, Martin-Perez and Villén (56) also reported such a study, in which they measured total proteome turnover in exponentially growing yeast and determined which parameters had the most influence on protein turnover rates. They determined that proteome turnover depended upon functional characteristics such as subcellular localization, membership of a protein complex, and gene ontology process more than it did on sequence-intrinsic or biochemical features and expression levels. Surprisingly, in contrast to the aforementioned study by the Selbach lab, they discovered a strong relationship between mRNA turnover and protein turnover rates (56). Further investigation is necessary to see if the different findings between studies like those of Martin-Perez and Villén (56) and Schwanhausser *et al.* (53) reflect genuine biological differences between yeast and mammalian systems.

Dynamic SILAC approaches have since been used in a variety of applications, including to determine how consistent protein half-lives are between different cell types. Although earlier studies found that orthologous proteins have conserved half-lives within different yeast and mammalian species (57, 58), with more closely related species displaying more similar protein half-lives (59, 60), Mathieson *et al.* (61) determined proteome-wide half-lives in 5 different nondividing primary cell types and found that protein half-lives from three human immune cell types (B cells, natural killer cells, and monocytes) were more similar to one another than they were to human hepatocyte and mouse neuron half-lives. A later study by the Aebersold group revealed that even culturing the same cell line over many passages can lead to similar distinctions in proteome turnover between stocks. In this investigation, Liu *et al.* (62) examined the degree of molecular and phenotypic variation in 14 different stocks of HeLa cells from 13 different laboratories around the world. After measuring genome-wide copy numbers, mRNA, protein, and proteome turnover for each cell line, they found substantial heterogeneity between samples and over the course of 50 passages of the same line. This may have been due to the genomic instability inherent in cultured cancer cells such as HeLa, resulting in varying degrees of aneuploidy across cell stocks, which affected gene expression at all levels. This study suggests important implications about using cultured cell lines in biological studies, as clearly culturing conditions change fundamental properties of cell stocks and subsequently the measurements of proteome turnover rates (62).

With the cell-type specificity of protein half-lives established, dynamic SILAC-based approaches can furthermore elucidate how proteome turnover relates to the ways that specific cell types perform their higher-order, specialized functions. For example, studies measuring proteome turnover rates in cultured neurons have identified mechanisms of proteostasis with direct implications for neurobiological processes such as memory formation and aging. The Ziv lab used dynamic SILAC to explore synaptic processes by measuring the half-lives of synaptic proteins and the influence of proteostasis on metabolic load. They found that protein turnover rates were not significantly different for presynaptic and postsynaptic proteins, or for proteins whose corresponding mRNAs have been found to localize to dendrites (63). Later work in neuronal cultures sought to determine which proteins are degraded by the UPS by identifying those whose half-lives increased upon proteasome inhibition. Although some proteins, including those related to glutamate receptor trafficking, were slowed by UPS inhibition, most synaptic proteins were not affected, indicating that they may be degraded by alternate pathways. They also found that inhibition of the proteasome also led to a profound blockage in the synthesis of a large number of synaptic proteins, indicating that there may be crosstalk between protein production and degradation pathways (64).

Additional studies in neurons found that not only the cell type but also the cellular microenvironment influences proteome turnover. Dörrbaum *et al*. (65) used dynamic SILAC to profile rat hippocampal neurons in neuron-enriched and glia-enriched cultures and found that proteins from glia cells had shorter half-lives than the same proteins in neurons. Moreover, they found that the presence of glia in co-culture changed the turnover of proteins in neurons. This indicates that while cell identity is an important determinant of protein turnover, turnover rates are also influenced by cell–cell contact and signaling.

A more recent study measured proteome-wide turnover using dynamic SILAC in both naïve and memory T cells. This study revealed that despite the quiescent state of naïve T cells, their proteomes are not inert, but rather contain a subset of highly turned over proteins, such as certain key transcription factors, that both help maintain the quiescent state of naïve T cells and facilitate a rapid transition into an activated state through their rapid depletion after stimulation. In addition, the authors found that, despite not being at all dependent on glycolysis, naïve T cells maintain high levels of glycolytic enzymes with very slow turnover rates, which allows naïve T cells to jumpstart glycolysis upon activation. With these data, the authors elucidated mechanisms by which the turnover rates of certain proteins are optimized in naïve T cells to prime them to efficiently exit quiescence after activation and maintain their new cell identity (66).

Proteome turnover analysis can be used to study disease states such as cancer and their potential treatments (67, 68, 69, 70, 71, 72, 73). One such study published by the Wiita lab examined proteome turnover in MM1.S multiple myeloma cells (72). They did not apply a global dynamic SILAC approach but rather targeted proteomics (selected reaction monitoring [SRM]) coupled to dynamic SILAC to acquire high-accuracy turnover data for 272 selected proteins. Owing to the high accuracy of the SRM measurements, the authors provided quantitative data for the heavy and light channel over time separately, and therefore estimated protein production and degradation separately. They compared the dynamic SILAC-determined protein synthesis data with ribosome footprinting data. Ribosome footprinting is a next-generation sequencing–based method to estimate the ribosome density on any given mRNA, and therefore is considered a good proxy for protein production (74, 75, 76). Indeed, in the unperturbed MM1.S cells, protein synthesis estimates from dynamic SILAC measurements correlated very well with synthesis estimates generated by ribosome footprinting. However, upon treatment with the drug bortezomib, a first-line chemotherapy drug and proteasome inhibitor, this correlation of synthesis estimates by dynamic SILAC and ribosome footprinting broke down. Many alterations in protein synthesis which could be seen by dynamic SILAC were not picked up by ribosome footprinting, underscoring that dynamic SILAC provides complementary and essential data that could be missed by ribosome footprinting, particularly under conditions of cellular stress (72).

Finally, dynamic SILAC can be used in whole-animal studies to elucidate *in vivo* proteome turnover rates by feeding animals isotopically labeled amino acids. This approach has been successfully applied to model organisms such as *Caenorhabditis elegans* (77, 78), *Drosophila* (79), zebrafish (80, 81), and mice (82, 83, 84). Fornasiero *et al.* (83) specifically measured proteome turnover in mouse brains, and through bioinformatic analysis, were able to characterize the pathway-, organelle-, organ-, and cell-specific effects on proteome turnover rates. Follow-up work examined the link between codon sequence and proteome half-lives using the same data set and found that codons with G or C bases at the wobble nucleotide position had longer protein half-lives than those ending with an A or U, although no causal link was established (82). Another *in vivo* dynamic SILAC study performed by Arike *et al.* (85) examined how proteome turnover in intestinal epithelial cells differed between normal and germ-free mice and across different segments of their intestines. They found that the median half-life of proteins is shorter in the small intestine than in the colon, and that proteins in germ-free mice typically have 1 day longer half-lives than proteins from conventionally raised mice. This result echoes *in vitro* studies that found cellular function and microenvironment are important determinants of protein turnover rates (65). However, when they looked at the half-lives of several long-lived proteins and a replication-dependent protein as a proxy for cell division rates, Arike *et al.* (85) actually found that the aforementioned median global half-life differences between the small intestine, the colon, germ-free mice, and conventionally raised mice were highly correlated with the differences they found in the estimated cell division rates. Nonetheless, ranked lists of protein half-lives can still elucidate meaningful differences in turnover rates of individual proteins between the cell types, which cannot be simply explained by differences in cell division rates, providing important insight about condition and cell-specific protein turnover.

An isotopic labeling strategy has recently been applied to study protein turnover in human ventricular cerebrospinal fluid from patients who had suffered a subarachnoid hemorrhage (86). The protocol was based on isotopically labeled leucine, a method dubbed whole-proteome stable isotope labeling kinetics, rather than the arginine- and lysine-based dynamic SILAC approach, but the experimental principle is the same. Lehmann *et al*. (86) detected proteins from multiple cell types, including neurons and immune cells, and found a link between protein turnover rates and the cell of origin, again echoing results from previous *in vitro* investigations. This approach could potentially be applied for biomarker discovery and indicates a potential crossing over of dynamic SILAC-like approaches from biology to medicine.

## Combinatorial Approaches to Determine Quantitative, Differential Proteome Turnover Data

Ideally, quantitative analyses of proteome turnover and protein half-lives include data on both protein synthesis and degradation rates separately. In conventional dynamic SILAC experiments, it is challenging to separate data for these two processes because of experimental variabilities in sample preparation and LC-MS/MS data acquisition (54, 55). Several different approaches have been implemented to specifically enable the determination of separate synthesis and degradation rates. One approach relies on the addition of an internal standard to the dynamic SILAC experiment (three-channel dynamic SILAC, Fig. 2), a method first used by the Lamond group in 2012 (54). This additional channel confers three advantages: it can be used for normalization, it provides information about protein abundance, and most importantly, it can be used to track protein synthesis and degradation separately (Fig. 2). Through this strategy, one can determine if protein and also the turnover rate changes are due to changes in protein synthesis rates, protein degradation rates, or the combined effect of both. Therefore, such an extended dynamic SILAC approach is best suited to characterize dynamic changes in these rates.

Indeed, this internal-standard approach has been applied during dynamic processes such as differentiation and activation of immune cells (25, 87). Kristensen *et al*. (87) found that over the course of the differentiation of C2C12 and THP1 cell lines, most proteins’ expression levels change because of differences in synthesis rates rather than degradation rates. Jovanovic *et al*. (25) expanded upon this approach when they examined the response of primary mouse dendritic cells to lipopolysaccharide using a multi-omics approach of three-channel dynamic SILAC and RNA-seq measurements. This method allowed for mRNA translation and protein degradation to be profiled independently, facilitating the modeling of dynamic rate changes upon dendritic cell activation. They found that although lipopolysaccharide-induced protein production changes were primarily driven by transcriptional changes, proteome remodeling of pre-existing proteins, often the so-called housekeeping genes, occurred at the level of mRNA translation and protein degradation. The same approach was used recently to examine proteome turnover changes during synaptic scaling, a type of homeostatic plasticity, in primary neurons (88). In this study, over half of the synaptic proteins in both presynapses and postsynapses showed changes in their turnover rates in different forms of synaptic plasticity, using different mechanisms to adjust turnover in upscaling and downscaling experiments.

The second strategy to measure protein production and degradation separately and track their changes upon perturbation is to combine dynamic SILAC with isobaric labeling (dynamic SILAC-TMT, Fig. 2) (55, 89, 90, 91). Combining isobaric labels and dynamic SILAC facilitates direct quantification of heavy isotope– and light isotope–derived peaks and also enables multiplexed analyses (55, 89, 90, 91). Tandem mass tag (TMT)-based quantification in particular allows a dramatic reduction of measurement time by combining up to 16 different samples in one LC-MS/MS run, while also reducing variation in peptides selected for measurement in data-dependent acquisition–based protocols (92). Savitski *et al.* (90) showed that such a combined approach enables the simultaneous analysis of changes in protein degradation and synthesis in a single mass spectrometric experiment of biological replicates subject to multiple treatment conditions, such as transcription factor inhibition, estrogen receptor modulation, and heat shock protein 90 inhibition. However, these approaches do incur additional reagent costs and require careful normalization (93).

The temporal resolution of dynamic SILAC approaches is limited by the metabolic activity of the target organism and the half-lives of its proteins. Although metabolically active organisms, such as bacteria, may resynthesize the majority of their proteins in hours, large, postmitotic eukaryotic cells have proteins with half-lives in the range of several days (25, 63, 65, 83). It is important to note that, in this context, the dynamic range of the mass spectrometer used for data acquisition provides a hard limit on the shortest time point that protein synthesis or degradation will be detectable. Poor ionization efficiency of a target peptide and coelution of highly abundant peptides may further confound detection (supplemental Table S1). It is thus important to always use appropriate control experiments and data analysis validation steps. A good starting point is to perform a mock dynamic SILAC incubation, that is, to treat an unlabeled sample as a dynamic SILAC sample in data analysis; in particular, for short time points, this control allows for the identification of false-positive and background signal levels (94). Conversely, the use of a fully heavy-labeled sample as a booster signal in combined dynamic SILAC-TMT protocols can increase detection of nascent peptides at early time points (94). This booster channel significantly increases the chance that heavy isotope MS1 peaks are selected for fragmentation (55, 89, 94), which subsequently increases the likelihood that low-abundant, newly synthesized, heavy-labeled peptides are quantified (94). Finally, reversed-SILAC channel experiments—in which heavy SILAC-labeled cells are pulsed with light SILAC amino acids as an additional experimental replicate—can help exclude signals inferred by confounding parameters such as isotopic envelope overlap (90).

A further consideration for dynamic SILAC experiments is that many primary cells require specific media and display significant sensitivity to medium changes—for example, neurons, which require conditioned media (supplemental Table S1). This poses a further challenge for analyses of short time points in these systems, as it would be impossible to tell if protein production rate changes at early time points are also affected or induced by the media change. Some groups bypass this issue by adding heavy amino acids in excess to the preconditioned media at time point zero (63) or make use of the preconditioned media that was already generated from a culture grown in heavy amino acids and therefore minimizes the adverse effects of the medium change on the cells (65, 88). These issues become even more challenging in whole-animal studies, where heavy amino acids are injected into or ingested by the animals. In contrast to cell-culture systems, the *in vivo* system does not get “flushed” by the number of heavy isotopes; therefore, heavy isotope incorporation will be slower. Moreover, the heavy isotopes may not enter all of the cells at equal rates, causing noisiness in early time-point signals (83). It is important to correct for these biases by carefully monitoring heavy isotope incorporation (84).

## Artificial Amino Acids Using Affinity Purification

The difficulties in detecting low-abundant, nascent proteins using pulse-only SILAC experiments are particularly salient in postmitotic cells. To overcome these challenges, the Schuman and Tirell labs developed bio-orthogonal non-canonical amino acid tagging (BONCAT) (95). BONCAT makes use of natural amino acid surrogates, typically methionine mimetics, that can be chemically targeted for purification. They usually carry an azido or alkyne functional group and can thus be immobilized on a solid phase using click chemistry and affinity purification. Although initially developed for neurobiological applications (95, 96, 97, 98), the technique has since been applied to multiple systems, including primary cells (99), tissue sections (98), and *in vivo* in a variety of organisms, including bacteria (100), archaea (101), plants (102), zebrafish (97), and other higher eukaryotes (103, 104). BONCAT can also be used to visualize overall proteome synthesis in cells using fluorescent non-canonical amino acid tagging (105) or to measure synthesis of target proteins in a spatially resolved manner using a proximity ligation assay (106).

Recently, artificial amino acid incorporation was genetically targeted to specific cell types using modified tRNA-synthetases (107) in living mice (108, 109), *Drosophila* (110) and zebrafish (111). This method enables specific analysis and imaging of the synthesis of a cell type–specific proteome in its physiological environment without prior cellular isolation.

The main challenge in click chemistry–based strategies is the biochemical purification of the labeled proteomes, as often only small fractions of the experimental sample are labeled and background adsorption to the affinity resin can be substantial (supplemental Table S1). This is particularly true for hydrophobic tissues such as brain lysates. These issues have been addressed through several different strategies, including covalent immobilization of nascent proteomes to enable stringent washing (99), or the use of cleavable crosslinkers to allow specific elution of labeled proteins (108, 109, 112, 113). An essential step to “quality-test” a workflow for nascent or cell type–specific proteome analysis is to perform a control experiment using methionine instead of azido-homo-alanine (AHA) or azido-nor-leucine (both are methionine analogs) and determine the experimental background proteome.

A logical extension of BONCAT was to combine it with a pSILAC approach. This approach improved the accuracy of nascent proteome analyses by the incorporation of heavy isotope–labeled amino acids in cell culture (114), macrophages (115, 116), and T cells (117). This combinatorial approach also improved the signal-to-noise ratio as the background will be dominated by peptides produced before the isotope pulse and can now be easily distinguished from the BONCAT-labeled proteins, which have to have the heavy isotope incorporated. The combined pSILAC and BONCAT labeling was also recently combined with TMT labeling to also provide the advantage of sample multiplexing (118).

Although the aforementioned studies used AHA labeling to look at production differences at shorter time windows or in specific cell types, this approach can also be reversed to study protein degradation, as demonstrated by the Selbach group (29). In this study, they applied a 1-hour AHA pulse followed by a cold methionine chase to NIH 3T3 cells for several different time periods. By combining this pulse-chase AHA experiment with SILAC labeling of samples with varying methionine chase lengths, they were able to precisely measure protein loss. This revealed that for ∼15% of proteins, protein degradation does not follow the predicted exponential decay function but rather undergoes a two-state model in which newly synthesized proteins are more likely to be degraded than older proteins (29). This process, dubbed “nonexponential degradation” (see also the “Definition of Terms and Turnover Rate Modeling” section), was found to be more common in subunits of complexes produced in superstoichiometric amounts (Fig. 3*B*).

## Integrative Multi-Omics Approaches

Above, we laid out tracer-based approaches that enable direct measurement of protein turnover parameters under steady-state and dynamic conditions. A potential alternative are multi-omics approaches using RNA levels, protein levels, and, optionally, ribosome density as measured through ribosome footprinting, which can be analyzed together to estimate protein synthesis and degradation rates. Owing to the lack of absolute protein estimates in steady-state proteomics measurements, these approaches work best on dynamic expression data, where relative changes can be very precisely measured on both RNA and protein levels (119, 120, 121, 122).

A good example of this type of integrative analysis is from Peshkin *et al.* (120) who looked at the mRNA-to-protein relationship during *Xenopus* embryonic development. The authors modeled protein synthesis and degradation by mass action kinetics and found that there are two major behavioral classes of proteins in the early embryo: one group with relatively stable expression levels, which were primarily inherited from the maternal cell, and a second group produced by the zygote that displayed greater “dynamicity” but lower abundance and had strong correlations with mRNA level changes. This indicates that proteome changes in early *Xenopus* development are primarily driven by changes in mRNA (120).

Another example of a multi-omics approach is from Eisenberg *et al.* (123) who used matched RNA-sequencing, ribosome profiling, and TMT-based proteomics to look at the temporal changes in gene expression during yeast meiosis. Here, ribosome footprinting was used as a proxy for protein production instead of direct metabolic labeling of the proteins themselves. As reported previously in other systems (124, 125, 126, 127), the authors found that members of the same protein complex showed stronger correlations with one another at the ribosome footprinting level than at the RNA level. However, by comparing the quantitative protein measurements with the ribosome footprint–based protein production proxies, they found that changes in protein levels of protein complex members matched one another significantly more closely than ribosome footprinting changes. Taken together, this implies that, although members of protein complexes can be synthesized at ideal stoichiometry (124, 125, 127), often they are synthesized at imprecise stoichiometry and their levels are adjusted by protein degradation (123). These results are very much in line with the aforementioned study by the Selbach group, where a subset of newly synthesized proteins was found to have significantly shorter half-lives than older proteins, leading to the conclusion that these proteins are members of protein complexes that are synthesized superstoichiometrically, and that excess proteins not incorporated in the protein complex are rapidly degraded (29).

The integration of multi-omics data has long been a major challenge because of differences in the instruments used to capture the data and the format in which it is generated. However, in the past few years, multiple computational tools have been developed that are relatively easy to implement and allow integration of such multi-omics time course data, estimating key regulatory parameter changes such as changes in protein synthesis and degradation (128, 129, 130, 131). One such ensemble of programs, protein expression control analysis (PECA) plus (129), can be used as a plugin in the popular “point and click” statistical software Perseus (132). Its various iterations can be used to calculate the probability for changes in mRNA or protein-level regulatory parameters at each time point in matched, large-scale time course data. Specifically, PECA-pS can determine synthesis and degradation rates from dynamic SILAC data, whereas the PECA core can be used to identify change points for protein-level expression and degradation using matched RNA and protein expression data (129). Programs such as PECA now also provide labs with limited computational experience the means to gain considerable regulatory insight from their gene expression data.

## Conclusion

Soon after the publication of the first dynamic SILAC publications, a review by Hinkson and Elias (1) outlined open questions in the field of proteome turnover. Among these were (1) cataloging the differences in protein turnover rates after activation, between cell types, and across species (2), understanding to what extent functionally and physically associated proteins are turned over in accordance with one another, and (3) matching proteins to degradation pathways (1). Although several of these questions have been addressed in selected model systems as outlined above, a comprehensive survey of biological systems is yet to be achieved. However, a few patterns regarding proteome turnover have emerged from the data that already exist.

One of the most robust findings across all of the studies surveyed here is that protein half-lives are similar for proteins found in the same complexes and that proteins known to participate in protein complexes have longer half-lives than those with no known association partners (54, 56, 61, 83, 85, 89). Complex turnover is not entirely coherent but typically subclustered based on the architecture of multimeric complexes, with more dynamic subunits showing higher turnover than stabilizing “core” subunits, such as has been seen for the proteasome (50, 61, 65, 123) (Fig. 3*B*). Additional mechanistic studies have revealed that for some complexes, certain subunits may be translated in excess and then degraded down to stoichiometric equivalencies (29, 123). The fact that members of large, multimembered protein complexes are more likely to have different turnover rates depending on subcellular location (54, 62) implies that these excesses of protein complex subunits may be generated to favor complex formation in one location distinct from the location where the complex functions (54). More generally, the relationship between complex membership and proteome turnover suggests the possibility of coordinated biosynthesis and degradation mechanisms for groups of interacting proteins (56, 63). These findings were discovered using different conditions and model systems, validating one another’s conclusions and suggesting that the tight coupling of protein turnover to complex membership is a basic feature of biological systems. Future studies could incorporate the use of size-exclusion chromatography (133, 134, 135, 136) with dynamic SILAC to better understand how proteins that are a part of several distinct complexes and/or are involved in “moonlighting” functions display variation in their turnover rates (137, 138). Additional robust findings include that subcellular localization (45, 54, 63, 65, 89, 139), proteoforms (89, 139), protein disorder (53, 56, 89), protein abundance (54, 83, 89), gene ontology category (53, 54, 56, 65), and cell type (61) are all correlated with proteome turnover rates to some degree (Fig. 3*A*).

In contrast to the aforementioned results that showed general agreement between several studies in different systems, there is a lack of strong agreement regarding the relationships between protein turnover rates and other key molecular features such as mRNA half-lives (53, 56, 120), N-terminal motifs (50, 54, 56, 65), codon sequences (82), amino acid composition (53, 56, 82) and other intrinsic properties. It is possible that this disagreement is due to biological differences in different systems. Large-scale meta-analyses and literature mining (140) of proteome turnover studies will be useful for reaching more answers, and potentially consensuses, particularly as more data are generated. In addition, re-examining pre-existing data sets with an eye toward unexplored or overlooked parameters will yield greater insight into the underlying biological and biochemical principles driving proteome turnover. A first glimpse into such studies is provided by recent work from the Ghaemmaghami lab. After re-examining a previously-published dataset reporting proteome turnover rates of fibroblasts derived from a variety of mammalian species, they proposed that differences in proteome turnover rates underlie organism-level phenotypes such as longevity, and that mechanisms of protein turnover regulation are linked to metabolic processes. As such, the principles underlying the protein turnover rate differences may vary substantially between species (60).

Proteome turnover studies are limited by two technical factors: sample complexity and dynamic range of detection. Sample complexity can be addressed by different fractionation techniques, improved chromatographic separation, and instrument and acquisition method development. However, although fractionation techniques such as high pH fractionation dramatically increase the number of analyzed peptides per sample, they also impose a limit on the study sample size because of the increase in LC-MS/MS measurement time, effectively multiplying the acquisition time by the number of fractions analyzed. Improvements in chromatographic separation, both in resolution and retention time stability, could significantly enhance peptide detection, fragmentation, and assignment (141). The detection of low-abundant, labeled peptide signals at, for example, short time points could be facilitated using targeted methods such as SRM, dramatically increasing sensitivity of a specific set of proteins at the expense of global proteome coverage (72, 142).

Instrument development in the past years has seen a shift in focus to ion-mobility separation. Although already a routine tool for hydrogen–deuterium exchange mass spectrometry analyses for a decade (143), ion mobility–based separation has only recently seen a surge of development with the trapped ion-mobility spectrometry time of flight–based parallel accumulation–serial fragmentation technique (144) and high-field asymmetric waveform ion-mobility spectrometry–based mass spectrometers (145). While technically based on different principles, both approaches achieve considerable separation power in the ion-mobility dimension and can thus help deconvolute complex spectra effectively. For example, parallel accumulation–serial fragmentation could enable selective accumulation and detection of short time-point, low-abundant, heavy-labeled isotope signals and high-field asymmetric waveform ion-mobility spectrometry could allow more efficient detection of these peptides because of a selective acquisition of specific charge states. Ion-mobility separation thus also improves the effective dynamic range that can be covered, as high-abundant, singly charged molecules/contaminants can be separated from proteolytic peptides carrying multiple charges. Together with data-independent acquisition strategies that recently outperformed data-dependent acquisition methods significantly in particular for low-input proteomics (146, 147), a significant increase in sensitivity could be achieved. Recent studies by the Aebersold and Liu labs already used a dynamic SILAC–data-independent acquisition approach to monitor proteome turnover at high accuracy and sensitivity (62, 73, 148).

For *in vivo* studies, the convolution of signals from different cell types still represents a major challenge. The use of artificial amino acids that are specifically incorporated into a target cell type only by genetic targeting may pose a very attractive option for studying proteome turnover in specific cell types in the future (100, 108, 111).

What comes next (Box 1)? A recent study by the Walther group is a great example of the kind of future studies that will shift us from more descriptive, correlative protein turnover studies to investigations that provide comprehensive functional insight into proteostasis and dynamic proteome remodeling. Building on previous work from the same group in which they compared the protein turnover rate between two distant yeast species (58), the authors used proteome-wide protein turnover measurements to match degradation pathways to individual proteins in *Saccharomyces cerevisiae* (58). To this end, they combined systematic single-gene deletions of over one hundred components of the yeast degradation machinery (*e.g.*, E2 and E3 ligases) with quantitative measurements of protein turnover, thereby mapping protein degradation pathways for hundreds of genes. This massive effort enabled the authors to identify the endogenous targets of the majority of E2 and E3 ligases and could serve as a blueprint for future studies about protein turnover (58). Owing to the technological advances described above, combined with the availability of genetic perturbation technologies such as CRISPR and targeted drug screens, the time is ripe to match protein turnover to functional pathways.

Box 1 **Some open questions and considerations regarding proteome turnover**

• What are the gene-specific regulators of protein production and degradation? For example, what are the targets of specific E3 ligases?
• What are the mechanisms by which synthesis and degradation pathways are coupled? How does inhibition of the proteasome change synthesis rates of many genes and vice versa?
• Through what molecular events are subunits of protein complexes coherently turned over?
• To what extent can we expect turnover rates to stay constant between two identical cultures? More importantly, how relevant are turnover rates functionally? Are differences in turnover rates between cultures predictive of differences in functional/phenotypic responses between them?
• What are the relative strengths and weaknesses of gathering turnover rate information compared with other cell parameters (*e.g.*, RNA-seq, proteomics, etc) in terms of giving relevant information that provides functional insight into the cell behavior and disease, beyond insight into the mechanisms of turnover itself?

These novel combinatorial approaches could be used to answer some of the remaining open questions regarding proteome turnover and its regulation (Box 1). Such questions include identification of the molecular events mediating the crosstalk between synthesis and degradation pathways (149) and illuminating the mechanisms by which complex assembly is coupled to protein turnover (150, 151). Future mechanistic studies could also elucidate how the determinants of protein half-lives, such as proteoform identity, are able to confer such variability in turnover kinetics (89). In recent years, enormous effort has been expended to quantify the proteomes of various cancer cell lines and primary tissues. We predict that similar efforts in categorizing proteome-wide protein turnover rates will be well worth the investment of cost and effort, as they will provide unique, complementary information regarding the principles underlying these essential processes driving protein expression (62).

## Conflict of interest

Authors declare no competing interests.

## Supporting Information

Supplemental Tables and References
