# Supplementary material for: Proteome Turnover in the Spotlight: Approaches, Applications, and Perspectives
Source: Mol Cell Proteomics. 2020 Dec 7;20:100016. doi: 10.1074/mcp.R120.002190 (PMC7950106; doi:10.1074/mcp.R120.002190)
Supplement: Supplemental Tables and References [file mmc1.pdf]

Supplemental Table 1: Advantages and limitations of approaches to study proteome turnover discussed in this review

Approach	Advantages	Limitations	Representative references
Biochemical analysis using radioactivity-, fluorescence- or tag-based detection	**Radioactivity**<br>• Wide choice of elements and compounds that can be used to incorporate tracers<br>• Cheap and easy to implement if appropriate lab setup is available<br>• Both bulk and targeted analyses can be performed	**Radioactivity**<br>• Toxicity of tracer elements<br>• Depending on radioactive tracer source, uptake can be mediated via multiple pathways, convoluting the readout<br>• Radioactive signal has no direct correlation with specific proteins – individual proteins can only be measured via purification or inferred via an orthogonal technique	Buus et al., 1994 (1)<br><br>Hara et al., 2004 (2)<br><br>Sangermann et al., 2001 (3)
	**Protein tagging**<br>• Targeted analysis of specific protein(s) of interest<br>• Sensitive detection of target protein<br>• Inexpensive and routine production<br>• Wide range of tags and strategies to choose from<br>• Can be used for both purification and detection (e.g. fluorescent protein constructs)	**Protein tagging**<br>• Addition of tag potentially compromises physiological localization, function, and turnover of protein target<br>• Addition of large tag particularly challenging for small and membrane proteins<br>• Analysis limited to one specific target protein at a time, and some tag types have limited capacity for multiplexing (e.g. fluorescent proteins)<br>• Blind to off-target effects	Yen et al., 2008 (4)<br><br>Eden et al., 2011 (5)
Chemical compounds to target synthesis and degradation	• Targeted analysis allowing isolation of either synthesis or degradation<br>• Wide range of compounds to choose from<br>• Usually strong phenotypes	• Off-target effects<br>• Cross-talk between synthesis and degradation can convolute results<br>• Compensatory effects from other pathways	Belle et al., 2006 (6)
Pulse only, mass spectrometry based, SILAC-derived strategies:<br>Dynamic SILAC (sometimes referred to as "pSILAC")	• Comprehensive, non-targeted analysis of proteome<br>• Tracers behave identically to natural amino acids, no modification of proteins required<br>• Broad applicability	• Media swap required for metabolic uptake, can induce cellular stress and convolute read-out<br>• Target systems with slow metabolism may show limited sensitivity for short time points due to low uptake<br>• Challenging for small polypeptides, membrane proteins or other proteins that produce only a limited number of proteolytic peptides or peptides with low ionization efficiency<br>• Challenging for extremely complex systems with high dynamic range.<br>• Challenging to acquire data on low-abundant proteins<br>• Due to amino acid recycling, heavy signal incorporation may be lower than the actual synthesis rate	Schwannhäuser et al., 2011 (7)<br><br>Ahmad et al., 2012 (8)<br><br>Mathieson et al., 2018 (9)<br><br>Jovanovic et al., 2015 (10)<br><br>Savitski et al., 2018 (11)

Pulse only, mass spectrometry based, SILAC-derived strategies "Continued"	**Dynamic-SILAC-TMT ("pSILAC-TMT")**<br>• Multiplexing of several experiments/samples<br>• Ability to determine synthesis and degradation rates separately<br>• Well-suited for direct comparison of synthesis and degradation rates if experimental design put the two experimental in the same TMT mix<br>• Can analyze overall synthesis using calibration channels<br>• Increase in data completeness compared to standard dynamic SILAC<br><br>**3-channel-dynamic SILAC ("3-channel-pSILAC")**<br>• Determine synthesis and degradation separately<br>• Analyze protein abundance changes	**Dynamic-SILAC-TMT ("pSILAC-TMT")**<br>• Ratio compression in TMT experiments<br>• Careful design of labelling schemes needed to avoid channel-based bias<br><br>**3-channel-dynamic SILAC ("3-channel-pSILAC")**<br>• Separate experiments required to obtain fully-labeled spike-in standards (for 3rd channel)	Dörrbaum et al., 2020 (12)<br><br>Salovska et al., 2020 (13)<br>(this paper also features an additional discussion about these different turnover measurement strategies)
Artificial amino acid tagging	• Specific analysis of newly-synthesized proteomes or cell type-specific proteomes; analysis of low-abundant proteins (e.g. BONCAT)<br>• Targeted study of sub-proteomes<br>• Can perform a true pulse-chase experiment that allows global measurements of protein half-lives by LC-MS/MS (see "pulse/chase-AHA-SILAC)<br>• Can be used for visualization of global proteome synthesis, e.g. FUNCAT<br><br>**pSILAC- BONCAT**<br>• Pulse experiments using artificial amino acids and selective purification of protein species produced in pulse window<br>Additional affinity purification of newly-synthesized proteins in pSILAC experiments allows for more sensitive detection<br><br>**"Pulse/chase-AHA-SILAC"**<br>• Direct detection of protein degradation in a "true" pulse-chase experiment	• Data on degradation only available if combined with SILAC<br>• Biochemical purification can be challenging for small target proteomes from complex hydrophobic tissue mixtures<br>• Careful background and purification efficiency controls required<br><br>**pSILAC- BONCAT**<br>• Uptake of artificial amino acids can be limited, prior cell starvation may be required<br>• Low incorporation of artificial amino acids into target proteins of interest (e.g. due to low Met-content)<br>• Artificial amino acid-based purification requires workflow optimization for each cell type<br><br>**"Pulse/chase-AHA-SILAC"**<br>• Cannot analyze protein synthesis<br>• Uptake of artificial amino acids can be limited, prior cell starvation may be required<br>• Complete SILAC labeling required<br>• Artificial amino acid-based purification requires workflow optimization for each cell type	Dieterich et al 2006 (14)<br><br>Landgraf et al., 2015 (15)<br><br>Schanzenbächer et al 2016 (16)<br><br>McShane et al., 2016 (17)<br><br>Alvarez-Castelao *et al.*, 2019 (18)

Integrative approaches	• Do not require special culture/growth conditions<br>• Robust, well-established protocols (e.g. RNA-seq)<br>• Comprehensive, optimized data analysis pipelines available	• Indirect inference of protein turnover from "indirect" proxy measurements<br>• Integration of different data modalities can be a significant challenge (e.g. count data vs. intensity data, limited overlap)	Peshkin et al., 2015 (19)<br><br>Eisenberg et al., 2018 (20)

References

1. Buus, C. L., Kristiansen, K., and Knudsen, J. (1994) Turnover of acyl-CoA-binding protein in four different cell lines measured by using two-dimensional polyacrylamide-gel electrophoresis. *Biochem J*. **297**, 555–560
2. Hara, H., and Shiota, H. (2004) Differential Increases in Syntheses of Newly Identified Trypsinogen 2 Isoforms by Dietary Protein in Rat Pancreas. *Exp Biol Med (Maywood)*. **229**, 772–780
3. Sangerman, J., Killilea, A., Chronister, R., Pappolla, M., and Goodman, S. R. (2001) α-spectrins are major ubiquitinated proteins in rat hippocampal neurons and components of ubiquitinated inclusions in neurodegenerative disorders. *Brain Research Bulletin*. **54**, 405–411
4. Yen, H.-C. S., Xu, Q., Chou, D. M., Zhao, Z., and Elledge, S. J. (2008) Global Protein Stability Profiling in Mammalian Cells. *Science*. **322**, 918–923
5. Eden, E., Geva-Zatorsky, N., Issaeva, I., Cohen, A., Dekel, E., Danon, T., Cohen, L., Mayo, A., and Alon, U. (2011) Proteome Half-Life Dynamics in Living Human Cells. *Science*. **331**, 764–768
6. Belle, A., Tanay, A., Bitincka, L., Shamir, R., and O'Shea, E. K. (2006) Quantification of protein half-lives in the budding yeast proteome. *PNAS*. **103**, 13004–13009
7. Schwanhausser, B., Busse, D., Li, N., Dittmar, G., Schuchhardt, J., Wolf, J., Chen, W., and Selbach, M. (2011) Global quantification of mammalian gene expression control. *Nature*. **473**, 337–342
8. Ahmad, Y., Boisvert, F.-M., Lundberg, E., Uhlen, M., and Lamond, A. I. (2012) Systematic Analysis of Protein Pools, Isoforms, and Modifications Affecting Turnover and Subcellular Localization. *Molecular & Cellular Proteomics*. 10.1074/mcp.M111.013680
9. Mathieson, T., Franken, H., Kosinski, J., Kurzawa, N., Zinn, N., Sweetman, G., Poeckel, D., Ratnu, V. S., Schramm, M., Becher, I., Steidel, M., Noh, K.-M., Bergamini, G., Beck, M., Bantscheff, M., and Savitski, M. M. (2018) Systematic analysis of protein turnover in primary cells. *Nature Communications*. **9**, 689
10. Jovanovic, M., Rooney, M. S., Mertins, P., Przybylski, D., Chevrier, N., Satija, R., Rodriguez, E. H., Fields, A. P., Schwartz, S., Raychowdhury, R., Mumbach, M. R., Eisenhaure, T., Rabani, M., Gennert, D., Lu, D., Delorey, T., Weissman, J. S., Carr, S. A., Hacohen, N., and Regev, A. (2015) Dynamic profiling of the protein life cycle in response to pathogens. *Science*. **347**, 1259038
11. Savitski, M. M., Zinn, N., Faelth-Savitski, M., Poeckel, D., Gade, S., Becher, I., Muelbaier, M., Wagner, A. J., Strohmer, K., Werner, T., Melchert, S., Petretich, M., Rutkowska, A., Vappiani, J., Franken, H., Steidel, M., Sweetman, G. M., Gilan, O., Lam, E. Y. N., Dawson, M. A., Prinjha, R. K., Grandi, P., Bergamini, G., and Bantscheff, M. (2018) Multiplexed Proteome Dynamics Profiling Reveals Mechanisms Controlling Protein Homeostasis. *Cell*. **173**, 260-274.e25
12. Dörrbaum, A. R., Alvarez-Castelao, B., Nassim-Assir, B., Langer, J. D., and Schuman, E. M. (2020) Proteome dynamics during homeostatic scaling in cultured neurons. *eLife*. **9**, e52939
13. Salovska, B., Zhu, H., Gandhi, T., Frank, M., Li, W., Rosenberger, G., Wu, C., Germain, P.-L., Zhou, H., Hodny, Z., Reiter, L., and Liu, Y. (2020) Isoform-resolved correlation analysis between mRNA abundance regulation and protein level degradation. *Molecular Systems Biology*. **16**, e9170
14. Dieterich, D. C., Link, A. J., Graumann, J., Tirrell, D. A., and Schuman, E. M. (2006) Selective identification of newly synthesized proteins in mammalian cells using bioorthogonal noncanonical amino acid tagging (BONCAT). *Proc. Natl. Acad. Sci. U.S.A*. **103**, 9482–9487
15. Landgraf, P., Antileo, E. R., Schuman, E. M., and Dieterich, D. C. (2015) BONCAT: Metabolic Labeling, Click Chemistry, and Affinity Purification of Newly Synthesized Proteomes. in *Site-Specific Protein Labeling: Methods and Protocols* (Gautier, A., and Hinner, M. J. eds), pp. 199–215, Methods in Molecular Biology, Springer, New York, NY, 10.1007/978-1-4939-2272-7_14
16. Schanzenbächer, C. T., Sambandan, S., Langer, J. D., and Schuman, E. M. (2016) Nascent Proteome Remodeling following Homeostatic Scaling at Hippocampal Synapses. *Neuron*. **92**, 358–371
17. McShane, E., Sin, C., Zauber, H., Wells, J. N., Donnelly, N., Wang, X., Hou, J., Chen, W., Storchova, Z., Marsh, J. A., Valleriani, A., and Selbach, M. (2016) Kinetic Analysis of Protein Stability Reveals Age-Dependent Degradation. *Cell*. **167**, 803-815.e21

18. Alvarez-Castelao, B., Schanzenbächer, C. T., Langer, J. D., and Schuman, E. M. (2019) Cell-type-specific metabolic labeling, detection and identification of nascent proteomes in vivo. *Nature Protocols*. **14**, 556–575
19. Peshkin, L., Wühr, M., Pearl, E., Haas, W., Freeman Jr., R. M., Gerhart, J. C., Klein, A. M., Horb, M., Gygi, S. P., and Kirschner, M. W. (2015) On the Relationship of Protein and mRNA Dynamics in Vertebrate Embryonic Development. *Developmental Cell*. **35**, 383–394
20. Eisenberg, A. R., Higdon, A., Keskin, A., Hodapp, S., Jovanovic, M., and Brar, G. A. (2018) Precise Post-translational Tuning Occurs for Most Protein Complex Components during Meiosis. *Cell Reports*. **25**, 3603-3617.e2
